# Symptom burden in patients with chronic rhinosinusitis with nasal polyps or global airway disease prior to biologic treatment: a qualitative study of patients' experiences

**DOI:** 10.3389/falgy.2025.1699186

**Published:** 2025-12-18

**Authors:** Christiane Haase, Lene Munch, Vibeke Backer, Bente Appel Esbensen

**Affiliations:** 1 Department of Otorhinolaryngology, Head and Neck Surgery, and Audiology, Rigshospitalet, Copenhagen University Hospital, Copenhagen, Denmark; 2 Centre for Health, University College Absalon, Roskilde, Denmark; 3 Department of Clinical Medicine, University of Copenhagen, Copenhagen, Denmark; 4 Rigshospitalet Glostrup, Copenhagen University Hospital, Glostrup, Denmark

**Keywords:** symptom burden, QOL, global airways, chronic diseases, corticosteroids

## Abstract

Chronic rhinosinusitis with nasal polyps (CRSwNP), is a prevalent inflammatory airway disease associated with type 2 inflammation. When CRSwNP coexist with asthma, the condition is referred to as global air disease (GAD). People how lives with CRSwNP or GAD often report a high symptom burden, for example persistent nasal obstruction, impaired smell and taste, disturbed sleep, fatigue, and reduced well-being. While these burdens have been quantified through clinical outcomes and patient-reported outcome measures (PROMs), there is limited qualitative research exploring how these peoples experience living with CRSwNP or GAD in their daily lives. The aim of the study was to describe the impact of CRSwNP or GAD on patients’ everyday lives before the initiation of biologic treatment.

**Methods:**

A descriptive qualitative interview study was conducted with 13 patients diagnosed with CRSwNP or GAD. Semi-structured interviews were analyzed using qualitative content analyzed as described by Graneheim and Lundman, resulting in the identification of thematic categories reflecting participants’ lived experiences.

**Results:**

Five categories were identified: (1) *feeling constantly unwell, like having a chronic cold*, describing the persistent sense of ill health; (2) *loss of senses disrupting everyday life*, illustrating how impaired smell and taste influenced both social and practical activities; (3) *illness leading to social isolation and emotional distress*, highlighting psychological and relational consequences; (4) *dependence on prednisolone—choosing between the lesser of two evils*, reflecting the balance between temporary relief and side effects; and (5) *Longing for a normal life*, expressing high expectations of biologic therapy as a potential turning point.

**Conclusion:**

This study showed that patients with CRSwNP or GAD experienced a substantial symptom burden, sensory loss, impaired work ability, and reduced social participation, all of which profoundly affected their daily lives. Many relied on prednisolone for temporary relief despite being aware of its serious side effects. The extensive impact of CRSwNP made participants hopeful that biologic therapy could provide more stable symptom control and help them regain a more “normal” life. CRSwNP or GAD is a multifaceted condition that affects patients’ emotional, psychological, and social well-being. These findings highlight the importance of adopting a holistic treatment approach, including consideration of psychosocial support to address the broader consequences of the disease.

## Introduction

Chronic rhinosinusitis (CRS) affects 5%–12% of the general population worldwide, thus making it a public health concern (1–4). CRS with nasal polyps (CRSwNP) has a significant impact on the quality of life (QoL) of people, affecting physical, psychological, social and occupation domains of QoL (2, 4, 5). When CRSwNP coexists with asthma, the condition is referred to as global airway disease (GAD). These patients often experience mood swings, irritability, frustration, loneliness, and helplessness (6). Patients describe the disease as “invisible”—a hidden disease—because there are no visible signs of the respiratory symptoms that they experience daily (5). They also report a lack of understanding, recognition, empathy, and support from the primary healthcare sector, society, and their families (5). Further, asthma often occurs as a comorbidity in patients with CRSwNP, with as many as 60%–70% with suffering from both conditions and many of these patients experience moderate symptoms, exacerbation, frequent shortness of breath, wheezing, chest tightness, and cough, all of which interferes with their day-to-day living, sleeping, and physical activity. In addition, patients often have frightening or unpredictable exacerbations of their condition (7).

CRSwNP or GAD is a multifaceted chronic condition that impacts patients’ emotional, psychological, and social well-being, and therefore requires a holistic approach to treatment. Continuous pharmacological treatment is necessary for CRSwNP or GAD; however, about one-third of patients experience poor disease control due to the modest effects of standard care. The main symptoms of CRSwNP include nasal blockage, facial pain/pressure, an impaired sense of smell, sleep disturbance or fatigue, and postnasal drip (2, 4). Increasing attention has been giving to the inflammatory association between the upper and lower airway—the global airways—highlighting the shared type-2 inflammatory mechanism (8, 9).

Biologic treatments have emerged as an add-on option for patients with inadequate response standard care. Standard treatment includes daily nasal irrigation, use of nasal, inhaled steroids for comorbid asthma and short courses of systemic steroids. Functional endoscopy can be performed if the outcome of standard care is unsatisfactory (2). Several three phase-III studies have demonstrated the positive effects in both patients with CRSwNP or GAD (10–12). However, biologic treatments are not yet standard in all countries, although current EPOS guidelines recommend biologics as a treatment option for patients with difficult to treat CRSwnP (2). Their potential to improve symptom and disease control and well-being is a driving advance in the management strategies (13, 14). Patients living with CRSwNP reported their concerns and satisfaction regarding future treatment with biologic (5).

According to the European Position Paper on Rhinosinusitis and Nasal Polyps (2) patients living with CRSwNP report an average symptom severity score of 8.2 on a Visual Analog Scale (VAS) ranging from 0 to 10 (2). Comparative studies reveal that CRS exerts a greater negative impact on QoL than other chronic diseases, including coronary artery disease, Parkinson's disease, and chronic obstructive pulmonary disease (2, 15, 16) Recently developed biologic drugs can substantially reduce the symptom burden and improve QoL (10, 11), highligtning the continued need for effective CRS management strategies (2, 17, 18). Despite advances in understanding and treating CRSwNP or GAD particularly with emerging biologic treatment, little is known about how patients experience and manage their lives with these conditions, especially prior to initiation of biologic treatments. While quantitative studies provide valuable data on symptom severity and treatment outcomes, they cannot capture the nuanced ways in which these conditions affect emotional well-being, daily functioning, and quality of life. A qualitative approach is therefore warranted to explore patients' lived experiences and expectations, providing critical insights supports more holistic and patient-centered care. Therefore, this study aims to describe the impact of CRSwNP OR GAD on patients' everyday lives before the initiation of biologic treatment.

## Method

### Design and setting

This study embraced a descriptive qualitative interview approach to describe patients' experiences of the impact of CRSwNP or GAD on their daily lives. The study was conducted at the Department of Otorhinolaryngology, Head and Neck Surgery, Rigshospitalet (RH), specifically within the Copenhagen Airway Clinic, which constitutes a single clinical unit. All interviews and data collection were carried out within this department. Also, the study adhered to the Consolidated Criteria for Reporting Qualitative Research (COREQ) (19) to ensure appropriate reporting.

### Participants and recruitment

To ensure information-rich selection (20), we employed a purposeful maximum-variation sampling strategy (21). Malterud's concept of information power (20) guided our considerations regarding sample size based on following four criteria aim, participants, quality of data and analysis strategy: 1) we assessed that the aim of the study was clear and was clearly defined, 2) we included relevant participants with a high degree of diversity in age, gender, and disease duration, representing a broad range of experiences within the topic investigated, 3) the interviews yielded high-quality data, with no new information emerging in the final interviews, and 4) content analysis, with its systematic analytic approach, confirmed the adequacy of our sample. Taken together, these factors support that our sample size was sufficient. The inclusion criteria for participants: 1) age ≥18 years; 2) diagnosis of CRSwNP (DJ324), GAD (DJ45); 3) referred the Copenhagen Airway Clinic, RH; 4) able to understand and speak Danish; and 5) planned initiation of biologic treatment soon after referral. The exclusion criteria were: 1) inability to provide inform consent and 2) inadequate Danish language proficiency to participate in an interview. These criteria ensured that participants represented the target population and could provide rich and relevant data. Patients were recruited from the Copenhagen Airway Clinic prior to the initiation of biologic treatment. An HCP from the clinic provided brief information regarding the study and asked whether the patient was interested in hearing more about it. If the patient agreed, they were verbally informed of the study's aim by CH (first author). Prior to the interviews, written and verbal informed consent were obtained from each participant. All the participants were assured that their data would remain anonymous and confidential. They were also informed of their right to withdraw from the study at any time, without any impact on their current or future treatment.

### Data collection

The interviews were conducted on the day of or a maximum of three days after the patients were screened for the initiation of biologic treatment (November 2023–February 2024). Prior to participation, patients received standardized information from the physicians about type-2 inflammation, indications, expected effects, potential side effects, national allocation principles to ensure equal access to biologics. The interview guide was inspired by the RSDI (Rhinosinusitis Disability Index) questionnaire: emotional, functional, and physical domains (22); the experiences of the research group; and feedback from three patients who were involved as research partners (23). The semi-structured interviews were conducted either face-to-face in the outpatient clinic or by telephone, depending on the participant's preference. This flexible approach was used to maximize participation and ensure that participants felt as comfortable as possible. Both type of interviews followed the same semi-structured guide to maintain consistency and ensure comparable data quality across both interview formats. CH conducted all the interviews and aimed to create a safe and stimulating interview situation by building a comfortable relationship with the participants (23). Additionally, CH asked follow-up questions to encourage participants to elaborate on their answers to obtain deeper insight into their daily lives living with CRSwNP or GAD. The following are a few examples of the questions asked: *I would like you to briefly tell me about the course of your illness? How do you experience your life now? How are you emotionally affected in your daily life? What challenges you most in your everyday life? What do you think treatment can do with regard to your symptoms and the control over your disease?* The interviewer audiotaped and transcribed the interviews verbatim, which were entered into NVivo15 (QRS International) for analysis.

To describe the characteristics of the participants’ symptom burden and disease management, all participants answered questions regarding asthma control (Asthma Control Questionnaire (ACQ-7) and Asthma Control Test (ACT), intensity of symptoms from (VAS for CRS, asthma, and loss of smell), and *quality of life* (SNOT-22) prior to the interviews (2, 24, 25) (see Table 1).

**Table 1:** Participants’ characteristics and demographics (*N* = 13).

Characteristic	Total (*n* = 13)
Chronic rhinosinusitis with nasal polyps (CRSwNP), *n* (%)	13 (100)
Asthma, *n* (%)	12 (92)
Atopy *n* (%)	10 (77)
Age, median (IQR)	55 [44–61]
Female, *n* (%)	5 (46)
Male, *n* (%)	8 (61)
Former smoker/never, *n* (%)	12 (92)
Current smoker, *n* (%)	1 (8)
Cohabitation status	
Living alone, *n* (%)	3 (30))
Duration of CRSwNP (years), median (IQR)	10 [6–20]
Duration of asthma (years), median (IQR)	10 [7–28]
Burden of symptom CRSwNP and asthma	
Asthma (ACT)^1^, median [IQR]	13.5 [10–17]
Assess symptoms and QoL of chronic rhinosinusitis (SNOT-22)^2^, median [IQR]	66 [60–88]
Asthma Control (ACQ-7)^3^, median [IQR]	2.8 [1.1–3.53]
Intensity of CRS symptom (VAS)^4^, median [IQR]	82 [74–89]
Intensity of loss of smell (VAS loss of smell)^5^, median [IQR]	100 [100–100]
Intensity of asthma (VAS Asthma)^6^, median [IQR]	70 [74–89]

1 ACT = Asthma Control Test. An ACT score of 20–25 indicates that the patient's asthma is well controlled, a score of 16–19 indicates that the asthma is partially controlled, and a score of 15 or lower indicates that the asthma is poorly controlled and that changes in treatment are needed (26).

2 SNOT = SNOT-22 is a questionnaire used to assess symptoms and quality of life related to sinonasal disorders. Patients were scored on 22 items on a 6-point Likert scale for assessing discomfort, ranging from 0 to 5, with a total score ranging from 0 to 110. The SNOT-22 score can be graded as “mild” when the SNOT-22 score ranges from 8 to 20, “moderate” when the score is in the range >20–50, and “severe” when the score is >50. Moreover, the scale has four subdomains: nasal symptoms, sleep disturbances, emotional/psychological distress, and aural/facial pain (24).

3 An ACQ is a standardised tool used to assess asthma control. A score of <0.75 on the scale indicates well-controlled asthma, while >1.5 indicates poorly controlled asthma. A change in the ACQ score of 0.5, which is considered the minimal clinically important difference (27).

4 VAS of CRS = visual analogue scale (VAS), ranging from 0 to 100. Tool used to measure the intensity of symptoms or discomfort. A score of 0 represents no symptoms (or no discomfort), while 100 represents the worst possible symptom (28).

5 VAS loss of smell = visual analogue scale (VAS), ranging from 0 to 100. Tool used to measure the intensity of symptoms or discomfort. A score of 0 represents no symptoms (or no discomfort), while a score of 100 represents the worst possible symptoms (28).

6 VAS of asthma = visual analogue scale (VAS), ranging from 0 to 100. Tool used to measure the intensity of symptoms or discomfort. A score of 0 represents no symptoms (or no discomfort), while 100 represents the worst possible symptoms (28).

### Data analysis

The interviews were analysed according to Graneheim and Lundman's qualitative inductive content analysis method (29). Consistent with this, the analysis was conducted using an open-minded iterative approach. First, CH, the second author LM, and the last author BAE read one interview and discussed the identified codes. Next, CH read the following interviews several times to get a comprehensive understanding of the data material. After CH read the transcripts line by line to identify meaning units consistent with the aim of the study. BAE and LM also read the transcript interviews and the condensed meaning units were discussed and labeled with codes. Fourthly, we compared the codes for similarities and differences and sorted into subcategories. In addition, because the differences between the subcategories had to be clear, we sought to keep the subcategories mutually exclusive. Lastly, we discussed the subcategories and, reached consensus on the final five categories. Each of the categories contained both the manifest content (description) and the latent content (interpretation) of the data (29). The developed codes, subcategories, and categories were consistently reviewed and compared against the raw data to ensure the validity and robustness of the analysis.

### Ethical considerations

All the data were pseudonymised and stored on an encrypted hard drive; the turnover key for the identification ID was placed on another closed hard drive. The study was conducted in accordance with the Declaration of Helsinki (30) and approved by the Danish Data Protection Agency **(p-2023-14575x**). Owing to the qualitative nature of the study, according to Danish law, no approval was required from a formal ethics committee (**F-23044487).**

### Patient involvement

We involved three patients as research partners in the study (two men and one woman with CRSwNP and or GAD). They provided feedback on both the written patient information and the interview guide. Their feedback was utilised constructively, thus leading to adjustments in the materials based on their input and resulting in the final version of the written patient information and the interview guide.

## Results

A total of 13 participants (five women and eight men) participated in this study; all of them were diagnosed with CRSwNP or GAD by an ear-nose-throat (ENT) specialist as well as a pulmonologist. The age range of the patients was 30–80 years [median (IQR), 55 (44–61)]. Three participants were interviewed face-to-face, and the remaining ten over the phone. The interviews lasted between 18 and 46 min (mean = 27 min). From the data, five categories were developed, which are presented below and supported by the selected quotations from the transcripts.

### Feeling constantly unwell, like having a chronic cold

Symptoms such as headaches, head or facial pressure, completely blocked nose, sneezing, and difficulty breathing, both generally and during physical activity, along with postnasal drip, left the participants feeling perpetually unwell. This was described as having a persistent common cold that would not go away, without seasonal variation. These symptoms had a negative impact on participants’ everyday lives, including their mental health, sleep quality, tendency for snoring, and ability to work. All participants reported experiencing a sense of inactivity, exhaustion, and fatigue, describing it as feeling out of sync, as if they were trapped in a “living prison” or a distorted version of themselves.

You walk around somewhat detached, as if beside your own life. I'm not saying it's time to push through, but it seems like you don't really want to. It's hard to explain, but it feels like you're not truly living when your mind isn't clear. Instead, it's just time slipping away. (Participant 10)

In their work lives, the feeling of having a persistent common cold reduced their productivity, requiring them to restart tasks they were in the middle of and adding pressure due to fatigue and forgetfulness. They adapted their work routines to manage their condition by reducing their working hours, working flexibly, or slowing down their work pace. One participant, a craftsman, had to carefully consider how to pack his toolboxes to carry them up and down from the basement to the third floor. Several participants mentioned that their working lives were becoming increasingly difficult, working full-time was challenging, and they often took sick leave or had to stop working and retire early due to ongoing health issues.

I simply can't take it anymore because I'm as tired as I already am. My body is simply not fresh. I don't have that energy anymore, so that's also one of the reasons why I've decided to retire. (Participant 4)

Symptom burden also affected sleep quality, with participants experiencing significant disturbances or a lack of restful sleep. For example, when trying to sleep, they felt that their bodies were seeking something other than the rest. Moreover, frequent awakenings at night due to experiencing of various symptoms, such as having to blow the nose due to nasal discharge while lying down, were common. Consequently, some chose to sleep sitting up or preferred to have their own bedroom to avoid disturbing their partner. Sleep disturbances lead to low energy, which impacts daily life. The participants did not feel like their “best selves” due to their cold-like symptoms and low energy levels. The participants also described a feeling of being overwhelmed by attending events and often chose to avoid them. They described how their daily lives had become rigid and overregulated because of the disease.

So, in that way, my life becomes very stuck. I feel that I don't have the energy I need, but that's because I use it and burn it off at work. (Participant 5)

### Disruption of everyday life due to loss of senses

Numerous participants experienced a significant impairment of their senses, particularly their sense of smell. A few of them reported a complete loss of smell, whereas others described diminished or distorted senses of smell and taste. These changes are often associated with feelings of grief and emptiness, thus evoking a profound sense of sadness over losing something deeply personal and cherished. The inability to smell loved ones, such as partners and children, or places, such as their own homes, had a life-altering impact on their well-being. Several participants realised that they had taken their sense of smell for granted before it was altered by the disease, which causes the swelling of nasal polyps that obstruct their nasal cavity. They reflected on missing simple yet profound scents such as the fresh smell of spring in the woods or the smell of autumn leaves decomposing on the forest floor.

Scents aren't something you think about in everyday life, that you’re conscious of in everyday life. But as soon as you don't have it, there are so many things that you usually appreciate that suddenly aren't there. I'm someone who loves walking in the woods. And smelling the scent of spring or smelling the change in weather that happens in autumn when the leaves are beautiful. (Participant 6)

Moreover, the loss of the sense of smell triggered a fear of being unable to detect dangers, such as fire, which could leave them unable to react in time. This concern extended to both professional and personal lives. Another concern was the risk of burning food while cooking, as they could not rely on their smell to monitor this event. Additionally, they mentioned that their impaired sense of taste made it difficult to know if food has gone bad, thus raising concerns regarding food safety.

The thing about being able to serve something to someone and not being sure if the food is spoilt or not because you can't smell it. So, I say that I think it's been bad. And it's made me sad .. I think it's been detrimental to the quality of life in every way. I mean, I'm such a foodie .. (Participant 6)

Losing their sense of smell deeply affected all the participants, and their emotional distress was evident during the interviews. They described how their lives had become duller and how they felt more depressed because of the loss of their sense of smell. The participants also shared experiences of embarrassment and feeling foolish in certain situations. One participant recounted forgetting a fresh mozzarella cheese in his workbag, which eventually exploded. Those around him noticed a bad smell and assumed that they stepped on something unpleasant. Unfortunately, he was unaware of the odour until he returned home, when his family brought it to attention. In addition, the participants described the inability to smell, taste, or enjoy food and wine prepared by their partner or friends as deeply frustrating and significantly diminishing their quality of life. Consequently, they avoided social events. For example, they no longer found pleasure in dining out with friends, as the food either seemed to lacked flavour or tasted the same, whether it was sushi, a liver pate sandwich, or a delicious steak. The taste and smell of alcohol were also affected. When the participants drank alcohol, they often experienced adverse reactions, such as watery eyes, sneezing, nasal congestion, or difficulty breathing due to intolerance to acetyl acid. They mentioned that drinking to socialise no longer felt as enjoyable as it had before the disease. They described this as an adjustment process, not only for themselves but also for those around them, who frequently forgot about their intolerance to alcohol. Consequently, the participants often found that they needed to explain their choice to order a soft drink instead of alcohol.

### Illness leading to social isolation and emotional distress

The participants described how symptoms—such as sneezing, itching, breathing difficulties, allergies, and fatigue—negatively impacted their social lives. They experienced a loss of close relationships and felt isolated due to the significant impact of the illness on their lives, as breathing difficulties can have profound social consequences. For example, the participants described a loss of intimacy with their partners, as they could no longer sleep together or enjoy leisure weekends in bed because they needed to sleep while sitting upright. They expressed that something vital was missing from their relationships. Moreover, weather changes also played a role and caused participants to avoid going outside in bad weather, as it worsened their sinus issues and left them feeling trapped and socially isolated.

It means that it all disappears. Work goes, friends go, family goes. It means that everything falls apart. The only option is for someone to come visit me here. And it used to be me who was out in the world. (Participant 3)

Impaired hearing was another condition experienced by a few of the participants; this caused them to feel both sadness and frustration, as they felt they were missing out on conversations in both their work and social lives.

My hearing has been severely impaired… I'm sad and frustrated because when I've talked to people.. (Participant 13)

The participants described how the disease made it difficult to juggle multiple tasks in both their work and personal lives, as the burden of symptoms had a significant impact. They felt that life had become limited and unmanageable, both socially and professionally, and reported a shift in their personalities from being happy and energetic to feeling drained. A couple of the participants also mentioned an impact on their roles as parents—for example, the symptoms of the disease affected the ability of parents to play football with their children.

I feel sad that I can't fulfil my role as a father. And not be present to give my children some memories and have the energy to play with them when they finally bother to play with me … So that's definitely what gets to me. Not fulfilling that role enough for them. It makes me sad. It makes me feel like I'm not good enough as a parent… (Participant 10)

Further, symptoms such as postnasal drip, nasal congestion, and fatigue led a few participants to withdraw from social life, thereby resulting in a sense of isolation. They highlighted the difference between voluntarily withdrawing from social activities and being forced to do so because of their symptoms. Choosing to opt out felt empowering, whereas being forced to withdraw due to their symptoms felt like being picked last for a football game, a reminder of how their social lives were affected. The disease also significantly limited their ability to participate in physical activities—such as skiing, diving, and running—both alone and in social settings. Many participants exercised far less than they would have liked because of breathlessness, coughing, fatigue, and low energy, with even moderate physical activity feeling overwhelming. Nasal obstruction and breathing difficulties were considered the most debilitating symptoms, and a few participants even experienced coughing during exercise, thus further reinforcing their feelings of inadequacy.

### Dependence on prednisolone—choosing between the lesser of two evils

For the participants, treatment with prednisolone was necessary from time to time to sustain daily life. It was common for them to be “addicted” to prednisolone and crave more. This was an effective and “fast fix” for the participants' high symptom burdens. One participant described himself as a “junkie”:

It sounds disgusting to say it. But it's like a junkie who needs a fix. Because you just know it works. So, at that point you have such a poor quality of life …(Participant 10)

Prednisolone was described as a “wonder drug” but also like a drug that “destroyed everything in its path”. The participants related how they were aware of the potential side effects of taking prednisolone—such as osteoporosis, “dry” bones, diabetes, hypertension, or thin skin with bruising—but that they still chose to use the treatment to give them a “break” due to their high symptom burden. For certain participants, the side effects worried them in one way or another, particularly because of thoughts about falling and breaking a leg if their bones had become fragile. Several patients had already experienced a few consequences of the disease, such as parchment skin and bruising, due to their intake of prednisolone. A few participants deliberately chose to switch between doctors (e.g., their general practitioner and ENT specialist) for the opportunity to get a prescription.

I wanted more of it because it made me feel amazing, but they [the doctors] said it was harmful to everything else. I guess that's why I couldn’t have it. Honestly, though, I didn’t care—I would’ve gladly given up 20 years of my life and dealt with the risks just to feel that good. (Participant 1)

Further, the participants could clearly feel when the effect of prednisolone was fading; for example, coughing again, nasal congestion, poor sleep, low energy levels, and moods that felt like “grey days when the sun does not shine”.

### Longing for a normal life—high expectations from biologic treatment

The participants had high expectations from biologic treatment before its initiation. They hoped to return to their normal everyday lives as they were living them before their illness and to regain the energy to be present again in their roles as parents, partners, friends, and work colleagues. They just wanted to live an untroubled existence. Several participants were emotionally touched by the prospect of a normal life.

I almost get completely euphoric at the thought that I can have a normal life again. (Participant 1)

They hoped that their sense of smell would return, either partially or completely. Dreams of being able to smell their partner or children again, taste and smell food, go out to eat delicious food were examples of what the participants hoped for. In addition, they hoped that the treatment would mean mornings without feeling half-sick or unwell. Several also saw it as the last opportunity to relieve their symptoms after many years of illness and many long-term attempts with other treatments without satisfactory symptom control. In contrast, a few of the participants also limited their expectations and hopes for biologic treatment because they were afraid of being disappointed if it turned out that it would not help their symptoms after all.

I try to keep my expectations low because I'm worried, I'll have a complete mental breakdown if it doesn't work out. (Participant 1)

## Discussion

In this qualitative study, which explored the experiences of patients living with CRSwNP or GAD, the participants emphasised the significant impact of their airway conditions and symptoms on daily life. They experienced years of living with upper and lower airway symptoms that profoundly affected their daily lives, including persistent chronic cold-like symptoms year-round, loss of senses, social exclusion, dependence on corticosteroids, and hope for change and maintenance of a better everyday life. Our study revealed that substantial symptom burden adversely impacted daily life, including work, social interactions, physical abilities, sleep, and overall well-being. Our findings uncovered that the participants often felt embarrassed or self-conscious in social and professional settings when nasal symptoms interfered with communication. Several also reported that the burden of CRSwNP or GAD negatively affected their mental health, leading to grief, depressive symptoms, and limitations in daily life. Previous research has reported similar findings, noting that the impact of CRSwNP or GAD can range from minor inconvenience to a significant reduction in QoL, thus affecting daily living as well (31, 32). Thus, these findings highlight the importance of HCPs in addressing and engaging in discussions during consultations regarding how CRSwNP or GAD affects various aspects of patients’ lives.

Various studies support the need for HCPs to be aware of the risk of depression and anxiety in patients living with CRS and asthma, and that they are often underdiagnosed and undertreated in clinical practice (33, 34). We also found that the participants experienced significant consequences of CRSwNP or GAD on their mental health, it might be meaningful for patients if HCPs address mental health concerns in the future, particularly during the initial consultation, to initiate a discussion regarding these issues. Such an approach would facilitate the early identification of potential concerns, thereby allowing HCPs to recognise and address these issues at an appropriate time. This emphasises the significance of addressing not only the physical symptoms of CRSwNP or GAD but also the psychological well-being of patients through multiple approaches.

We also found that sleep deprivation due to CRSwNP or GAD leads to significant morning fatigue and impaired sleep quality. Consequently, participants reported feeling exhausted not only in the morning but throughout the day, which affected their ability to function at work, within the family, and in the social context. Similar findings have been reported by several prior qualitative studies—for example, sleep disturbances negatively affect concentration and work performance; however, these issues are often overlooked in patient care (5, 15, 31). Other studies on patients with type 2 inflammatory diseases—including CRSwNP or GAD—have also described how fatigue, sleep disturbances, and reduced concentration had significant consequences for patients' daily lives, as also revealed in the current study (5, 15, 31).

Thus, it might be important for HCPs to not only address issues directly related to CRSwNP or GAD but also be aware of sleep-related issues and how these might impact patients' productivity at work, their role as partners or parents, and their overall mental well-being. One possibility could be to address optimal sleep hygiene, such as maintaining a sleep diary and ensuring better adherence to pre-bedtime treatment, such as nasal douching and the use of nasal and inhaled steroids to improve sleep quality (35, 36).

Another key finding of this study was the profound impact of sensory dysfunction—particularly in smell, taste, and hearing—on participants' daily lives and psychological well-being. The loss created a sense of disconnection, disrupting their sense of normality and reducing their engagement in activities, such as eating, socialising, and performing everyday routines. Two separate qualitative studies also found that sensory loss, particularly smell and taste, can lead to feelings of isolation and disconnection, thereby negatively impacting mental well-being and contributing to anxiety, which aligns with the current findings (5, 31). These studies further reported that loss of sensory input diminished the enjoyment of socialising, eating, cooking, and other food-related activities (5, 31). This aligns with the current participants’ descriptions, where sensory dysfunction was not only a persistent but also a significant contributor to psychological distress, including an increased risk of anxiety and depression. Considering this, it highlights the need for HCP to recognise the profound impact of sensory loss on patients’ mental well-being and daily life. Thus, it is important for HCPs to initiate conversations that acknowledge the social and emotional consequences of sensory dysfunctions. A prior retrospective observational study supported the idea that early identification may enable patients to adopt coping strategies, such as maintaining social connections to reduce the risk of poor mental health outcomes (37). Furthermore, a previous systematic review and meta-analysis suggested that therapeutic approaches, including olfactory training, could be incorporated into treatment plans, as this method has shown promise in partially restoring olfactory function (38). Importantly, treatment adherence is essential to achieve optimal therapeutic outcomes. While sensory loss had an impact on participants’ daily lives, challenges related to medical treatment also emerged. The participants expressed ambivalence regarding the use of prednisolone, but they were often willing to endure severe side effects in exchange for even brief relief from symptoms. They often described a deep longing for a sense of normalcy, even if only temporary, despite being fully aware of serious risks, such as osteoporosis, diabetes, and bone fractures. This aligns with two prior qualitative studies that also found that patients with CRSwNP or GAD expressed ambivalence toward prednisolone, reporting a range of adverse effects including anxiety, depression, insomnia, and weight gain (5, 15). In the present study, while a few patients experienced symptom relief, others questioned the efficacy and voiced concerns regarding long-term use and dependency. This illustrates that the level of desperation patients may feel and highlights the urgent need for alternative treatments. Therefore, it is essential that HCPs strike a balance between the patient's need for symptom relief and the risks associated with prednisone dependence and its side effects.

Biologic treatments have demonstrated promising improvements in QoL and overall well-being in patients with CRSwNP or GAD (11, 39). Building on this, we aimed to explore not only how patients with CRSwNP or GAD describe living with a chronic condition but also their expectations regarding the initiation of biologic treatment. All participants expressed that the prospect of beginning biologic treatment gave them a strong sense of hope for regaining a normal life, such as recovering their sense of smell and no longer feeling constantly ill. However, this hope was tempered by cautious expectations as many feared the possibility of disappointment. These nuanced insights into the multifaceted burden of CRSwNP or GAD may help inform more holistic, multidisciplinary, and patient-centered care approaches, emphasizing the need to address emotional and social well-being alongside symptom control. Therefore, it would be meaningful for future research to further explore the impact of biologic treatment on the disease.

### Methodological considerations and limitations

To ensure the trustworthiness of our research, the concepts of credibility, transferability dependability, and conformability were used (29). One aspect of credibility included participants who lived with CRSwNP or GAD and who wanted to talk about it. Although, we aimed to sample participants with variation in age, sex, and disease duration, we were not able to achieve a fully balanced sample. More men than women were included, which to some extent reflects the higher prevalence of CRSwNP among men. In addition, the duration of disease varied considerably among participants. We acknowledge these limitations, but do not consider them to compromise the overall credibility of the study. A key methodological strength of this study was its focus on patients' lived experiences of CRSwNP or GAD prior to biologic treatment, providing in-depth insight often is overlooked in quantitative research. Credibility was further strengthened though the involvement of the three patient research partners with CRSwNP or GAD in the study to ensure that the interview guide reflected their perspectives and experiences. Unfortunately, we were not able to involve them in other tasks within the project. Although, their engagement contributed to opportunities for establishing accountability, relevance, and transparency in the interview guide and research (40, 41). The flexibility of the interview guide facilitated an in-depth exploration of relevant topics that were raised during the interviews. Furthermore, we explained and described the selection of the participants, data collection, and analysis in detail, which enabled us to determine whether the results are transferable to other contexts.

Further, our findings provide insight into how patients with CRSwNP or GAD experience their everyday lives before the initiation of biologic treatment. Dependability was ensured though semi-structured interviews with different supportive questions for each topic. The first author, CH, was the only author who conducted the interviews and had a pre-understanding of patients living with CRSwNP or GAD. CH attempted to put her pre-understanding aside by being as neutral as possible during the interviews and analysed and interpreted the results in a transparent manner. However, in the data analysis, two of the co-authors (LM and BAE) contributed with additional clinical experience and pre-understanding, which ensured a more nuanced approach. Confirmability was supported by collaborative review of the manuscript and findings among all authors. Another strength is our use of Lundman and Graneheim's content analysis method and following their outline steps (29) in our analysis approach. Following their method provided us with the opportunity to go back and forth among data, codes, and categories and, consequently, get closer to the participants’ everyday experiences of living with CRSwNP or GAD before the initiation of biologic treatment.

## Conclusion

In this study, we aimed to describe the impact and burden of CRSwNP or GAD on patients' daily lives before the initiation of biologic treatment. An essential finding was that the participants experienced high symptom burden, sensory loss, disrupted work, and consequences related to social interactions and family activities, all of which had a huge impact on their acceptance of their everyday lives. The extensive consequences of CRSwNP or GAD led the patients to take prednisolone to obtain temporary relief, even though they were fully aware that it was associated with serious side effects. Moreover, the substantial impact of CRSwNP or GAD on participants' daily lives made them hope for a future treatment—for example, biologic treatment—that could provide more stable relief from their symptoms and help them lead a more “normal” life. Finally, recognising the need to incorporate psychosocial support into treatment plans is essential to address the broader social and emotional consequences of the disease. Future research should investigate how psychosocial support, and multidisciplinary approaches can be effectively integrated into treatment strategies, with particular attention to their organization within healthcare systems and their long-term impact on quality of life and disease management.

## Data Availability

The datasets presented in this article are not readily available due to patient confidentiality. Requests to access the datasets should be directed to the corresponding author.
